# Decoding TikTok: Utilizing Social Media for Difficult Conversations About Ageism

**DOI:** 10.1093/geroni/igab046.738

**Published:** 2021-12-17

**Authors:** Lauren Bouchard

**Affiliations:** Concordia University Chicago, Chicago, Illinois, United States

## Abstract

Understanding ageism is a key aspect of gerontological curriculum. Media examples (e.g., television and movies) can be effective tools, and yet gerontological educators should stay updated on new media trends to encourage student interest. This presentation will explore a new social media application (i.e., TikTok) to help students recognize and dismantle their own ageist beliefs. The presenter will describe and explain the classroom activity, instructions for finding and downloading content, as well as the social media application itself. In this activity, students brainstorm their preconceived notion of older adults to catalyze open discussion regarding societal beliefs. Next, a few video examples, with both positive and negative portrayals of older adults are presented for discussion. Students may also bring other examples for participation credit to this class. This symposium presentation will include an interview activity guide, additional breakout group instructions, and other tips for creating impactful class discussion on ageism.

